# scHiMe: predicting single-cell DNA methylation levels based on single-cell Hi-C data

**DOI:** 10.1093/bib/bbad223

**Published:** 2023-06-10

**Authors:** Hao Zhu, Tong Liu, Zheng Wang

**Affiliations:** Department of Computer Science, University of Miami, 330M Ungar Building, 1365 Memorial Drive, Coral Gables, 33124-4245, FL, USA; Department of Computer Science, University of Miami, 330M Ungar Building, 1365 Memorial Drive, Coral Gables, 33124-4245, FL, USA; Department of Computer Science, University of Miami, 330M Ungar Building, 1365 Memorial Drive, Coral Gables, 33124-4245, FL, USA

**Keywords:** single-cell DNA methylation prediction, single-cell Hi-C, graph transformer

## Abstract

Recently a biochemistry experiment named methyl-3C was developed to simultaneously capture the chromosomal conformations and DNA methylation levels on individual single cells. However, the number of data sets generated from this experiment is still small in the scientific community compared with the greater amount of single-cell Hi-C data generated from separate single cells. Therefore, a computational tool to predict single-cell methylation levels based on single-cell Hi-C data on the same individual cells is needed. We developed a graph transformer named scHiMe to accurately predict the base-pair-specific (bp-specific) methylation levels based on both single-cell Hi-C data and DNA nucleotide sequences. We benchmarked scHiMe for predicting the bp-specific methylation levels on all of the promoters of the human genome, all of the promoter regions together with the corresponding first exon and intron regions, and random regions on the whole genome. Our evaluation showed a high consistency between the predicted and methyl-3C-detected methylation levels. Moreover, the predicted DNA methylation levels resulted in accurate classifications of cells into different cell types, which indicated that our algorithm successfully captured the cell-to-cell variability in the single-cell Hi-C data. scHiMe is freely available at http://dna.cs.miami.edu/scHiMe/.

## INTRODUCTION

DNA methylation in mammals is a biological process in which a methyl group is attached almost exclusively to the cytosine. It regulates gene expression by blocking the binding of transcription factors to DNA or by recruiting the proteins involved in gene repression [1]. Many cellular processes, including embryonic development, X chromosome inactivation and chromosome stability maintenance, are related to DNA methylation [2]. Reduced representation bisulfite sequencing (RRBS) is the technique that has been widely used to detect DNA methylation levels on bulk cells. Identifying the genomic regions with different methylation levels in bulk cells can differentiate standard and aberrance tissue types [3–5]. In contrast to biological assays, DNA methylation levels of bulk cells can also be predicted by computational models, which saves time and effort for wet-lab experimentalists [6–8].

The chromosome conformation capture (3C) technology was developed to reveal the spatial proximities between a single pair of genomic loci in a cell population [9]. After that, 3C-On-Chip (4C) [10], 3C-Carbon Copy (5C) [11] and Hi-C techniques [12] were developed to capture spatial proximities between a locus and all other genomic loci, between all restriction fragments within a given genomic region and between all possible fragments across the whole genome in a cell population, respectively. Particularly, the Hi-C technique reveals topologically association domains (TADs) as the structural and functional units of the genome [13, 14] and makes it possible to classify TADs into families [15]. The Hi-C data can also be used to computationally reconstruct the three-dimensional (3D) structure of the chromosomes [12, 16, 17].

Unlike the standard or bulk Hi-C technique that captures the genome-wide spatial interactions from a population of cells, the recently developed single-cell Hi-C technique can capture the spatial proximities of the whole genome of the individual cells and can be used to reveal cell-to-cell variability [18]. Like the single-cell Hi-C experiment, the single-cell reduced representation bisulfite sequencing (scRRBS) [19] technique can detect the DNA methylation of single cells. Hui *et al*. [19] used scRRBS to obtain the methylation information of 1 million CpG sites of individual diploid mouse and human cells at single-base resolution.

A new experiment protocol named single-nucleus methyl-3C sequencing was recently developed that simultaneously detected both single-cell DNA methylations and the single-cell spatial proximities of the genomes [20]. Moreover, Li *et al*. [21] reported a molecular assay approach named Methyl-HiC that simultaneously captured the chromosomal conformations and DNA methylations in individual mouse cells. However, not all biological wet labs are capable of performing this protocol. Therefore, there are limited data sets available that contain simultaneously captured single-cell methylation data and single-cell Hi-C data.

The sparsity of the single-cell methylation and Hi-C data is a challenge for computational methods. A frequently used approach to enhance the data of a single cell is by aggregating the data from other similar cells or building a meta-cell. In the research of [22–24], when a cell has insufficient methylation values, it integrates the methylation data from the neighboring cells. Uzun *et al*. [25] also used the idea of meta-cells when predicting single-cell gene activity levels based on single-cell methylation data.

## MATERIALS AND METHODS

### Overview

**Figure 1:**
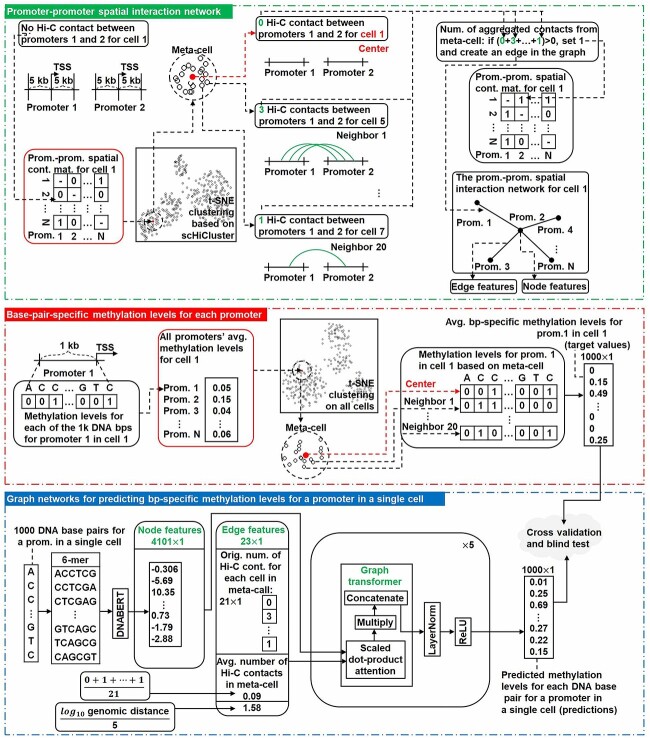
The overview of the architectures of scHiMe-promoter. The top area illustrates the process of building promoter–promoter spatial interaction networks. The middle area shows how the true DNA methylation values, or the target values for the machine learning algorithm, are defined based on meta-cells. The bottom area shows the architecture of the graph transformer including the illustrations of node and edge features.

Figure 1 shows the overview of the methodologies of scHiMe when it is used to predict bp-specific methylation levels of all of the promoter regions genome-wide (referred to as ‘scHiMe-promoter’). The methodologies of scHiMe when it is applied on promoters together with the corresponding first exon and intron regions (scHiMe-exon1) and randomly selected regions in the whole genome (scHiMe-whole-genome or scHiMe-wg) are the same as this except the target regions are changed. This and the following sections will elaborate on the methodologies of scHiMe-promoter, and the detailed methodologies of scHiMe-exon1 and scHiMe-whole-genome can be found in the supplementary document.

The single-cell Hi-C data of the target cell were used to generate a promoter–promoter Hi-C contact matrix for the target cell. The idea of meta-cell was then applied, with which the promoter–promoter Hi-C contact matrices of the neighboring cells were aggregated to generate the aggregated promoter–promoter Hi-C contact matrix for the target cell. Based on the aggregated promoter–promoter Hi-C contact matrix, a promoter–promoter spatial interaction network was constructed. The true base-pair-specific DNA methylation values or target values for the 1000 base pairs in the target promoter were also generated based on meta-cell. Node features and edge features were generated and input into the graph transformer network, which contained five blocks of graph transformers. Details of each step will be presented in later sections.

### Data sets

The first data set used in this research contains the simultaneously-captured single-cell Hi-C data and DNA methylation data from the brain cells of a 29-year-old male caucasian [20]. The cells in this data set are from 14 different cell types, and we only kept and used nine cell types (Astro, ODC, Ndnf, Sst, Vip, Endo, L23, MG and OPC) that contained at least 30 cells. We used the cells in the Astro, ODC and Ndnf cell types to build the training data set, cells in the Sst and Vip cell types for the validation data set and the cells in Endo, L23, MG and OPC cell types for the blind-test data set. Details about our quality controls can be found in the supplementary document. This first data set was also used to benchmark the performance of classifying cells into different cell types based on the predicted single-cell methylation levels of promoters.

The second data set is similar to the first but from a 21-year-old male caucasian also from the research [20]. We picked the same four cell types: Endo, L23, MG and OPC, and applied the previously mentioned quality-control criteria. This data set was only used for blind testing and clustering cells into cell types based on the predicted single-cell methylation levels of promoters.

We also benchmarked a version of scHiMe, in which we still used the cells in Astro, ODC and Ndnf as a training data set and cells in Sst and Vip as a validation data set, but used the cells in Endo, L23, MG, OPC, L4, L5, L6 and Pvalb for the blind-test data set.

Furthermore, we mixed the Astro, ODC and Ndnf cells from both data sets 1 and 2 as the training data set, similarly for the validation data set (Sst and Vip) and blind-test data set (Endo, L23, MG and OPC), to test the performance of using mixed data extracted from different persons (referred to as ‘scHiMe-promoter-mixed’).

We used another human single-cell Hi-C data as our third data set, which was from [26]. We used three cell types: K562, HAP1 and HeLa, and selected 100 cells from each cell type that had the highest number of single-cell Hi-C contacts. Since single-cell methylation data were not included in this data set (the authors only performed single-cell Hi-C experiments), we only used it for benchmarking the performance of classifying cells into cell types based on the predicted methylation levels of promoters. The number of cells used for each scHiMe predictor can be found in supplementary Table S1.

The transcription start site (TSS) definitions used in this research were downloaded from the GENCODE human annotation version 19 [27].

### Building meta-cells for single-cell Hi-C data

Due to the sparsity of the single-cell Hi-C data of a target cell, we combined the target cell and other cells, which share similar Hi-C patterns with the target cell, into a meta-cell and used the single-cell Hi-C contacts of the meta-cell as the Hi-C contacts for the target cell. For the cells used for training and validation, their cell types were considered known information. Therefore, the similar or neighboring cells used to build each meta-cell were selected from the same cell type as the target or central cell. Details can be found in the supplementary document.

### Building meta-cells for single-cell DNA methylation data

Due to the sparsity of the single-cell methylation data, we combined the target cell with other neighboring cells, which share similar methylation patterns with the target cell, into a meta-cell and used the aggregated single-cell methylation levels of the meta-cell as the methylation levels for the target cell. Details can be found in the supplementary document.

### Promoter–promoter spatial interaction network

In the promoter–promoter spatial interaction network, each node represents a promoter. The aggregated Hi-C contacts from meta-cells were used to define edges in the promoter–promoter spatial interaction networks. An edge was created between two promoters if at least one aggregated Hi-C contact exists between the two promoter regions. The promoter region used here was defined as the genomic region that contains 5 kb upstream and downstream of the TSS plus the TSS of a gene.

### Node and edge features

The node features were the encodings of DNA nucleotide sequence for each 1-kb promoter/node, which was generated by DNABERT [28]. Specifically, we generated the 6-mer representation (4-mer and 5-mer were also tested, see supplementary Table S2) for each base pair in the DNA sequence and input the representations of all base pairs into DNABERT, which outputted 4101 real numbers as the codings of the promoter. For the promoters existing in the negative strand, we used their complementary sequences in the positive strand in the order of 5’ to 3’ (this 5’ to 3’ is in terms of the positive strand) as the sequences that were input to DNABERT.

The first edge feature contains 21 integers, which are the numbers of Hi-C contacts between the two promoters that the edge connects. These Hi-C values are from the target cell and its 20 neighboring cells in the meta-cell. The second edge feature contains one integer, which is the average number of occurrences of nonzero Hi-C contacts in the meta cell. An example is given in the supplementary document. The third one is the genomic distance between the two promoters. In total, the edge feature for each edge consists of 23 values.

### Graph transformer

The node features that are input to the $o$-th block of the graph transformer are $P^{(0)}=\{{p^{(o)}_{1},p^{(o)}_{2},...,p^{(o)}_{n}}\}$, where $p$ stands for a promoter, and $n$ is the number of promoters. Based on the node features, a query vector for the $i$-th node feature is defined as $q^{(o)}_{h,i}=W^{(o)}_{h,q}p^{(o)}_{i}+b^{(o)}_{h,q}$, where $h$ indicates that it is the $h$-th head in the block, and $W^{(o)}_{h,q}$ and $b^{(o)}_{h,q}$ are the trainable parameters for the query vector.

Similarly, a key vector for the $j$-th node feature is defined as $k^{(o)}_{h,j}=W^{(o)}_{h,k}p^{(o)}_{j}+b^{(o)}_{h,k}$.

The edge features input to the transformer block are represented as $E^{(0)}=\{{e^{(o)}_{1},e^{(o)}_{2},...,e^{(o)}_{n}}\}$. Specifically, the edge feature for the edge between node $i$ and node $j$ after the $h$ head-attentions is encoded as $e^{(o)}_{h,ij}=W^{(o)}_{h,e}e^{(o)}_{ij}+b^{(o)}_{h,e}$, where $e^{(o)}_{ij}$ is the original edge features between node $i$ and node $j$, and $W^{(o)}_{h,e}$ and $b^{(o)}_{h,e}$ are trainable parameters.

The encoded edge features are added to the key vector for calculating the multi-head attention: $\alpha ^{(o)}_{h,ij}=\frac{\left \langle{q^{(o)}_{h,i},k^{(o)}_{h,j}+e^{(o)}_{h,ij}}\right \rangle }{\sum \nolimits _{u\in \mathcal{N}(i)}\left \langle{q^{(o)}_{h,i},k^{(o)}_{h,j}+e^{(o)}_{h,ij}}\right \rangle }$, where $\left \langle{q,k+e}\right \rangle $ is the exponential scale dot-product that is calculated as ${\left \langle{q,k+e}\right \rangle }=exp(\frac{q^{T}(k+e)}{\sqrt{d}})$ with $d$ is the hidden size of each head attention.

After the above $h$-th head attention is completed, a value vector is calculated as $v^{(o)}_{h,j}=W^{(o)}_{h,v}p^{(o)}_{j}+b^{(o)}_{h,v}$, where $W^{(o)}_{h,v}$ and $b^{(o)}_{h,v}$ are trainable parameters.

Message aggregation is performed to integrate the value vectors of all the radius-one neighboring nodes, each of which is labeled as $j$, to node $i$ as $\hat{p}^{(o+1)}_{i}=\big |\big |^{h}_{1}\sum \nolimits _{j\in \mathcal{N}(i)}\alpha ^{(o)}_{h,ij}(v^{(o)}_{h,j}+e^{(o)}_{h,ij})$, where $\big |\big |^{h}_{1}$ is the concatenation operation that is performed on all $h$ head attentions.

Different from the paper [29], we directly applied the normalization layer and ReLU layer to the multi-head attention as follows, which, based on our evaluation, resulted in a better performance (results of comparisons not shown): $p_{i}^{(o+1)}=ReLU(LayerNorm(\hat{p}_{i}^{(o+1)})$.

Also different from the paper [29], which only aggregated head attention for the node features, we also aggregated the head attention for the edge features as follows, which resulted in better performance (results of comparisons not shown): $\hat{e}^{(o+1)}_{ij}= \big |\big |^{h}_{1}(\alpha ^{(o)}_{h,ij},e^{(o)}_{ij})$.

A normalization and ReLU layer are added to get the edge features that will be input to the next block: $e^{(o+1)}_{ij}=ReLU(LayerNorm(\hat{e}^{(o+1)}_{ij}))$.

For the last block, the concatenation operation on all $h$ head attentions is replaced by the average on multi-head output as: $p^{(o+1)}_{i}=\frac{1}{H}\sum _{h=1}^{H}\big [\sum \nolimits _{j\in \mathcal{N}(i)}\alpha ^{(o)}_{h,ij}(v^{(o)}_{h,j}+e^{(o)}_{h,ij})\big ]$.

### Training, validation and blind test

The total number of graphs used for training is 21 758 for scHiMe-promoter (the numbers for other scHiMe predictors are shown in supplementary Table S3). The graph transformer that we used was modified based on the transformer_conv in the torch_geometric.nn.conv pack [29], which was published in [30]. We tested different values of the number of transformer blocks including 3, 5, 7 and 10, and different values of head attentions including 1, 2 and 5, and found the number of blocks of 5 and the number of head attentions of 1 resulted in the best performance on the validation data set, which was used in our final model. The other details can be found in the supplementary document.

### Evaluation

#### Naive predictors

Since there is no other published tool to compare performance, we built two naive predictors that make predictions directly based on the methylation levels of the neighboring nodes in the promoter–promoter spatial interaction networks. The details about these naive predictors can be found in the supplementary document.

#### Performance metrics

To benchmark the performance of scHiMe, we calculated the Pearson’s correlation (PCC), Matthew’s correlation (MCC), average precision (AP) and area under the curve (AUC) of the receiver operating characteristic curve (ROC) between the predictions and ground truths. Our evaluations were performed only on the base pairs that had true methylation levels available.

We used the predicted methylation levels on all the cytosines and guanines in CpGs because guanines in the positive strand indicate cytosine in the negative strand. When we refer to the methylation values of guanines in this paper, we mean the cytosines on the negative strand.

#### Cell classification and its evaluation

The predicted methylation levels on all the CpGs of all promoters were input into the K-means clustering algorithm with the number of clusters set to four. To evaluate the clustering results, or evaluate whether the groupings of cells in the clustering results match the true cell types, we applied the adjusted rand index (ARI) to measure the similarities between the clustering results and the true cell types. We named this type of ARI score ARI-all-data. A close to zero ARI score indicates random labeling, and the ARI score of one indicates that two clusterings are identical.

To visualize the predicted methylation levels on all the CpGs of all promoters, we input the data into the t-distributed stochastic neighbor embedding (t-SNE) algorithm, and the top two components were obtained, which were treated as the X and Y coordinates for plotting the cells in a two-dimensional (2D) space. Another type of ARI score was calculated based on the clustering results of these top two components, which was referred to as ARI-two-components in this paper. Details about the two ARI scores can be found in the supplementary document.

#### Importance of individual CpGs in promoters for clustering cells

We used the random forest algorithm from the sklearn package [31] to measure the importance of individual CpGs in promoters for classifying cells. Random forest was used to classify a cell into one of the four cell types: Endo, L23, MG and OPC. We used the predicted methylation levels on all of the CpGs, including both cytosines and guanines, from all of the promoters genome-wide as the features. More details can be found in the supplementary document.

#### Cell clustering based on housekeeping genes

Housekeeping genes are typically constitutive genes that are required for the maintenance of basic cellular functions. We downloaded the human housekeeping genes from the HRT Atlas database [32]. Among all the housekeeping genes that we downloaded, 1967 of them matched the genes that were used to define the promoters in our research and were used in this analysis. We randomly selected another 1967 promoter from all of the promoters that we had in the research and used them as the baseline analysis.

#### Collective Influence algorithm

We applied the collective influence (CI) algorithm [33] to the promoter–promoter spatial interaction networks built on data set 2. A list of influencers ranked from the most significant to the least significant influencers was outputted from the CI algorithm for each cell. Details can be found in the supplementary document.

## RESULTS

### Blind test results on data set 1 and data set 2

Table 1 shows the evaluation results for the predictions from scHiMe-promoter and scHiMe-promoter-mixed based on five evaluation metrics. We can find that the overall absolute difference between the scHiMe-promoter-predicted and true methylation levels is 0.100 on average, and the AUC of the ROC for scHiMe-promoter is above 0.93 on both data sets. The graph transformer trained and tested on mixed data from two different persons also achieved similar performances. Moreover, the performances of both scHiMe-promoter and scHiMe-promoter-mixed are much better than the performances of the two naive predictors, which indicates the contributions of the graph transformer in terms of capturing graph topology and data patterns in the networks. The performances of scHiMe-promoter on each of the Endo, L23, MG and OPC cell types are shown in supplementary Table S4.

**Table 1:** The evaluation results for scHiMe-promoter, scHiMe-promoter-mixed and two naive predictors on the blind-test cells on data sets 1 and 2. scHiMe-promoter was tested on 10 repeated runs with the standard error provided.

Prediction	Data	PCC	Average	MCC	AUC	Average
method	set		precision			abs. error
scHiMe-promoter	1	0.740$\pm $0.021	0.777$\pm $0.090	0.705$\pm $0.004	0.934$\pm $0.002	0.100$\pm $0.002
scHiMe-promoter	2	0.725$\pm $0.020	0.776$\pm $0.088	0.696$\pm $0.002	0.931$\pm $0.002	0.101$\pm $0.002
scHiMe-mixed	1 and 2	0.723	0.768	0.701	0.934	0.097
Naive 1	1	0.059	0.189	0.058	0.533	0.286
Naive 1	2	0.097	0.203	0.095	0.550	0.274
Naive 2	1	0.485	0.377	0.479	0.813	0.154
Naive 2	2	0.545	0.441	0.530	0.836	0.148

Table 2 shows the evaluation results for scHiMe-exon1 and scHiMe-whole-genome. Overall, predicting only promoter regions achieved the best performance (Table 1), whereas predicting randomly selected regions of the whole genome had slightly worse performances. Using chromosome 1 as an example, the number of nodes/promoters in the graph of chromosome 1 for scHiMe-promoter is 2021 but is increased to 4840 for scHiMe-whole-genome, resulting in 21 758 training examples/graphs (each graph is a training example) for scHiMe-promoter and only 8468 training examples/graphs for scHiMe-whole-genome (see supplementary Tables S3 and S5) due to the limit of CPU memory of our server. We believe the less amount of training examples of scHiMe-whole-genome is the cause of the slightly worse performance.

**Table 2:** The evaluation results for scHiMe-exon1 and scHiMe-whole-genome on the blind-test cells on data sets 1 and 2.

Prediction	Data	PCC	Average	MCC	AUC	Average
method	set		precision			abs. error
scHiMe-exon1	1	0.713	0.737	0.682	0.929	0.101
scHiMe-exon1	2	0.703	0.734	0.671	0.919	0.103
scHiMe-whole-genome	1	0.645	0.850	0.612	0.853	0.182
scHiMe-whole-genome	2	0.650	0.850	0.616	0.859	0.185

The results in Tables 1 and 2 are based on the blind tests performed on Endo, L23, MG and OPC. The benchmarking results on eight cell types: Endo, L23, MG, OPC, L4, L5, L6 and Pvalb are listed in supplementary Table S6, showing similar performances.

**Figure 2:**
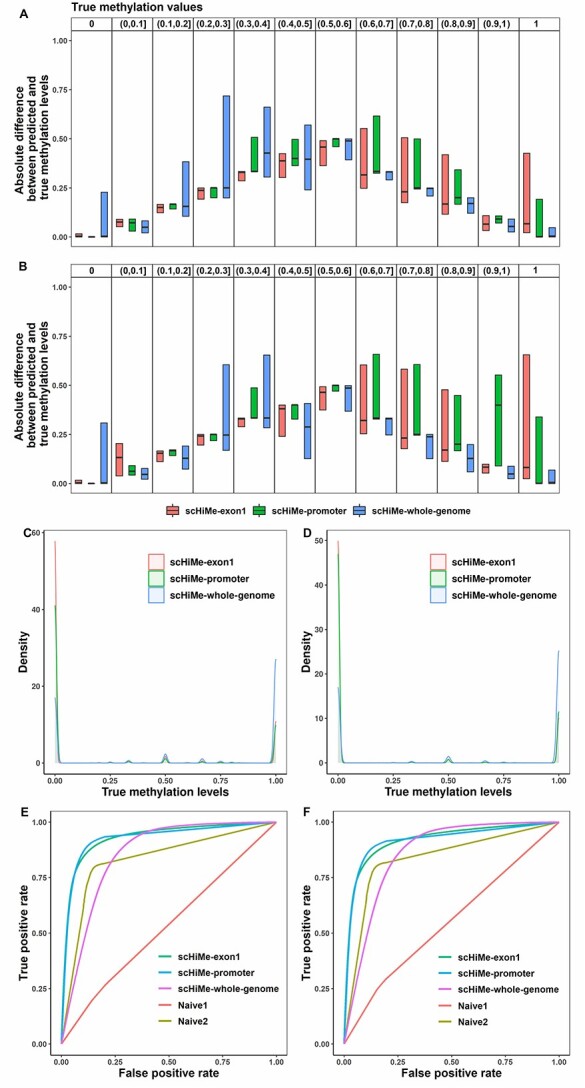
A-B. Absolute differences between the scHiMe-predicted and true methylation levels on the blind-test cells in data sets 1 and 2, respectively. C-D. Distributions of the true methylation levels on data sets 1 and 2, respectively. E-F. ROC curves and the AUC scores for the predictions of scHiMe and two naive predictors on the blind-test cells in data sets 1 and 2, respectively.

To further analyze the performances of scHiMe, we plotted the absolute differences between the predicted and true methylation levels for different ranges of the true methylation levels, see Figures 2A and 2B for data sets 1 and 2, respectively. We can see that for all the predictors when the true methylation levels are 0 and 1, the absolute differences reach the minimum with mean values close to 0, whereas larger absolute differences are found when the true methylation levels are in the range of (0.5, 0.6]. Note that Figures 2C and 2D show that almost all of the true methylation levels are at 0 and 1, which are 77.9% and 18.5% for data set 1, and 78.9% and 19.3% for data set 2, respectively. In reality, a DNA base pair in a single cell can either be methylated or unmethylated corresponding to either 1 or 0 as the ratio of methylated reads divided by total reads. Therefore, the non-0-or-1 ratios or levels are the cases caused by the noise or imperfections in the methyl-3C experiments. However, we still train our predictors to predict real numbers instead of conducting binary classifications as we believe that the ratio somehow may be used to indicate the confidence of being methylated or unmethylated. We also plotted the absolute differences between the true methylation levels and the predicted levels of two naive predictors, see supplementary Figure S1. The ROC of the predictions of scHiMe and two naive predictors are shown in Figures 2E and 2F for data sets 1 and 2, respectively.

### Cell-type classification based on the predicted methylation levels

**Figure 3:**
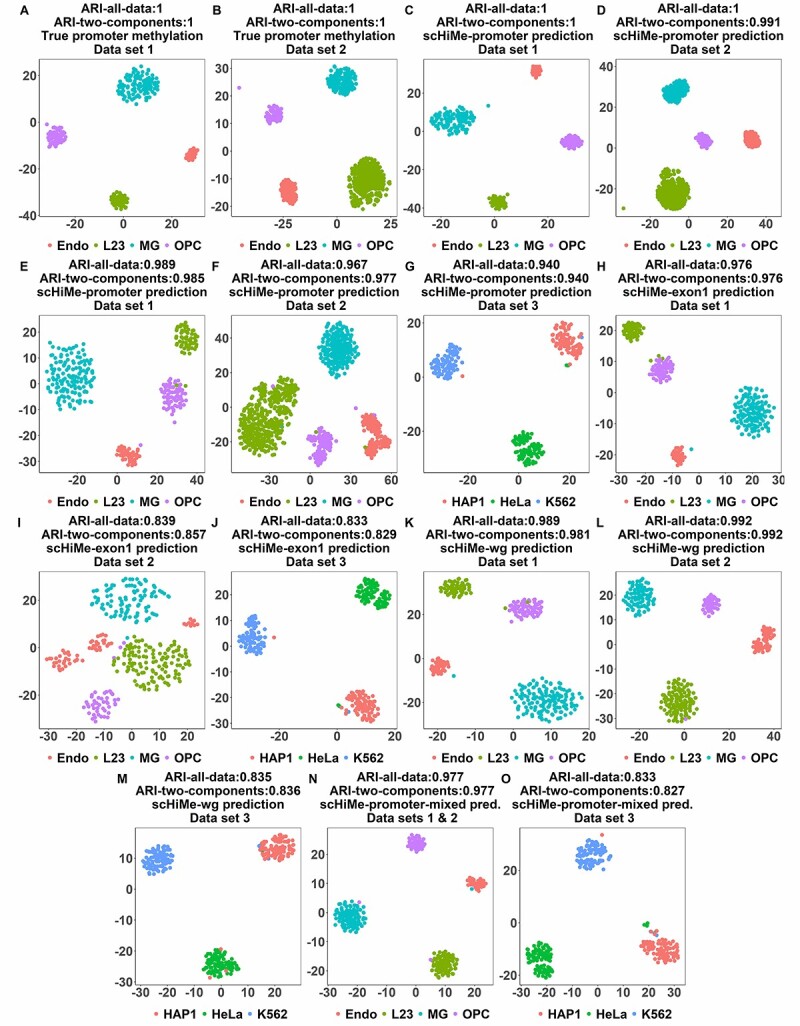
A. Visualization of the top two components from t-SNE based on true methylation levels on data set 1. For the Cs and Gs of CpGs that did not have methylation data available, -1 was used. B. Similar to A, but on data set 2. C. Visualization of the top two components from t-SNE based on scHiMe-promoter-predicted methylation levels on data set 1. For the Cs and Gs of CpGs that did not have methylation data available, -1 was used. D. Similar to C, but on data set 2. E. Visualization of the top two components from t-SNE based on scHiMe-promoter-predicted methylation levels on all Cs and Gs of CpGs no matter whether true methylation levels are available or not. F. Similar to E, but on data set 2. G. Similar to E, but on data set 3. H. Similar to E, but for scHiMe-exon1. I. Similar to F, but for scHiMe-exon1. J. Similar to G, but for scHiMe-exon1. K. Similar to E, but for scHiMe-whole-genome. L. Similar to F, but for scHiMe-whole-genome. M. Similar to G, but for scHiMe-whole-genome. N. Similar to E, but for scHiMe-promoter-mixed and data sets 1 and 2 mixed. O. Similar to G, but for scHiMe-promoter-mixed on only data set 3.

Figures 3A and 3B illustrate the first two components from t-SNE based on the true methylation levels on data sets 1 and 2, respectively. For the cytosines and guanines of all CpGs of all promoters, if there are true methylation levels available, the true methylation levels were used for the cytosines and guanines; and if not available, -1 was used. It can be found that the cells were clustered almost perfectly to their real cell types, which matched the results of a similar experiment in [20].

Figures 3C and 3D are similar to Figure 3A and 3B, but the scHiMe-promoter-predicted methylation levels were used for the cytosines and guanines of CpGs that have true methylation levels available. For the cytosines and guanines of CpGs that did not have true methylation levels available, -1 was still used. It can be found that almost all of the cells were still correctly classified. This demonstrates that the predictions from scHiMe maintain the unique patterns of single-cell methylation data for each cell type.

For Figures 3E and 3F, we used the scHiMe-promoter-predicted methylation levels for all of the cytosines and guanines of CpGs, no matter whether the true methylation levels were available or not. This experiment tested whether scHiMe-promoter-predicted methylation levels resulted in the correct classification of cells even for the cytosines and guanines of CpGs that did not have true methylation data available from the wet-lab experiments. Figures 3E and 3F demonstrate that almost all of the cells were still correctly classified into partitions, which further demonstrated the promising accuracy of scHiMe-promoter.

Figure 3G is similar to Figures 3E and 3F, but is on data set 3, which is another independent data set. It can be found that scHiMe-promoter achieved similar performance on this data set.

Figures 3H-O show the clustering results for scHiMe-exon1, scHiMe-whole-genome and scHiMe-promoter-mixed with similar successful performances.

We also tested clustering cells based on scHiMe-promoter-predicted methylation levels of individual promoters and then ranked the promoters based on the ARI-all-data scores (see supplementary Table S7). We found that using only the first-ranked promoter resulted in an ARI score of 0.664 and using the top 32 promoters brought the ARI score to 0.898, for details see supplementary Figure S2A.

We further clustered cells based on scHiMe-promoter-predicted methylation level of each cytosine or guanine of a CpG on data set 2. It can be found that when the top 28 cytosines or guanines were used, the ARI score reached 0.572 and remained there even when 1000 cytosines were used, see supplementary Figure S2B for details.

All clustering results mentioned above were benchmarked on Endo, L23, MG and OPC. The results for clustering cells on eight types: Endo, L23, MG, OPC, L4, L5, L6 and Pvalb are shown in supplementary Figure S3. It can be found that the scHiMe-promoter-predicted methylation levels on L4, L5, L6 and Pvalb do not result in the successful clustering of the cells in these four cell types. We believe the reason is that the single-cell Hi-C data of the four cell types cannot lead to successful clustering of the cells based on another study, see Figure 5c in [12]. As single-cell Hi-C data are one of the two input features of the graph transformer and the other feature, which is the reference DNA sequences, for all the cells are the same, it is not surprising that the predicted DNA methylation levels cannot successfully classify the cells.

### Performance from the perspective of network degree

**Figure 4:**
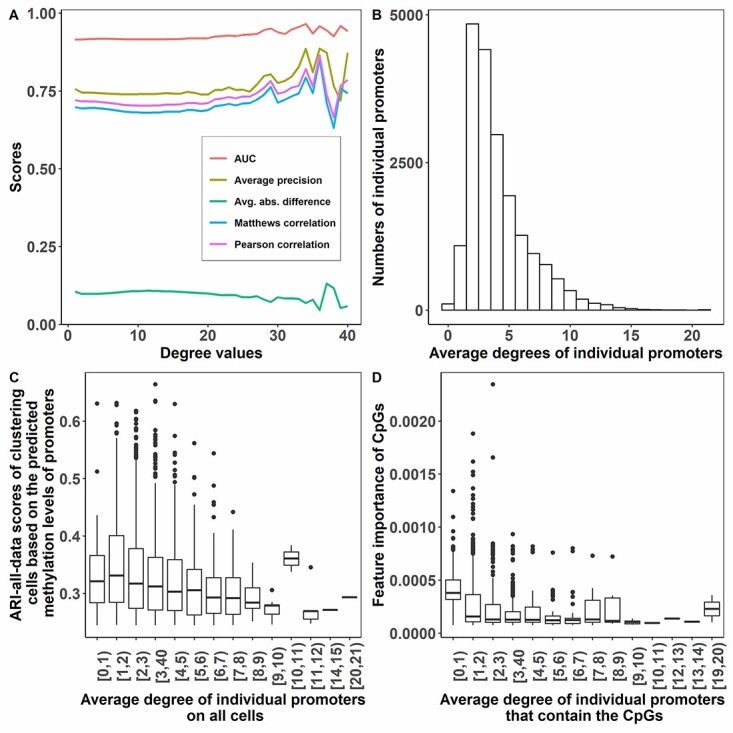
A. Evaluation matrices for different degree values on data set 2. B. Distribution of average degree for all of the promoters on data set 2. C. ARI-all-data scores of clustering cells based on the predicted methylation levels of the individual promoters having different average degrees. D. Feature importance for all cytosines and guanines of CpGs from the individual promoters having different average degrees.

The degree of a promoter in a promoter–promoter spatial interaction network was defined as the number of edges that connected to the promoter. Figure 4A shows the evaluation metrics for different degree values from 1 to 40 on data set 2. It can be found that the performances are stable when the degree is from 1 to 20, and the performances slightly increase and then vary when the degree is larger than 20. The best AUC 0.965 is with degree 34, and the best Pearson’s correlation, Matthew’s correlation coefficient, average precision and average absolute difference, which are 0.864, 0.853, 0.886 and 0.045, respectively, are with degree 36.

Figure 4B illustrates the average degree distribution for all of the promoters on data set 2, which shows that most of the promoters have an average degree of 2 and 3.

We further tested the performances when using the individual promoters having various average degrees to cluster cells. Figure 4C shows the ARI-all-data scores when using the individual promoters of different average degrees to cluster cells. The promoters used in Figure 4C were the top 3000 promoters ranked by the ARI-all-data scores when each of them was used to cluster cells. It can be found that the promoters with average degrees between 10 and 11 are associated with the highest ARI scores although it needs to be noticed that few promoters fall into the degree range of [10, 11). Other than that, the promoters in the degree range of [1, 2) resulted in slightly higher ARI scores when used to cluster cells into different types. In general, the increase in the average degree values is associated with the decrease in ARI scores. Supplementary Figure S4 shows the same plot but generated on all promoters, which leads to similar findings.

We gathered the feature importance generated by random forest for all cytosines and guanines of CpGs, and then visualized the feature importance concerning the degrees of the promoters that contained the cytosines and guanines, see Figure 4D. Only the top 3000 cytosines or guanines that have the highest feature importance values were used for the analysis in Figure 4D. Interestingly, the higher the average degree value is, the lower mean feature importance the cytosines and guanines have.

We also benchmarked the performance of scHiMe from the perspective of network influencers but did not find a significant relationship between the significance of influencers and the evaluation metrics. Moreover, we did not find a better performance of clustering cells when using more significant network influencers, see supplementary Figure S5.

### Cell clustering and feature importance for housekeeping genes

**Figure 5:**
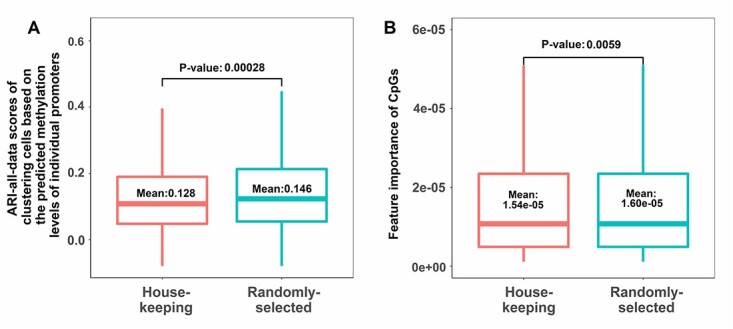
A. ARI scores of using scHiMe-promoter-predicted methylation levels of housekeeping genes and randomly selected genes to classify cells. The P-value between the ARI scores in the two groups is shown in the figure. B. Feature importance of the cytosines and guanines of the promoter CpGs for housekeeping genes and randomly selected genes when their scHiMe-promoter predictions were used to classify cells. The P-value between the ARI scores in the two groups is shown in the figure.

Housekeeping genes are the genes that are required for maintaining basic cellular functions, which are essential for the existence of cells. Housekeeping genes usually are considered to be expressed in all cells with stable expression levels. Therefore, if the scHiMe-promoter predictions are accurate, the predicted methylation levels on the promoters of housekeeping genes should be less effective in distinguishing cell types compared with the promoters of baseline genes. Figure 5A shows the ARI-all-data scores when housekeeping genes and randomly selected genes were used to classify cells. The mean of ARI scores for using housekeeping genes is 0.128, which is lower than the ARI score for using randomly selected genes, which is 0.146, with a *P*-value (student t-test) of 0.0028. Figure 5B shows the feature importance of cytosines and guanines of CpGs when housekeeping genes and randomly selected genes are used, which have the means of $1.542 \times 10^{(-5)}$ and $1.601 \times 10^{(-5)}$, respectively, with a P-value of 0.0059. These results further proved that the predictions from scHiMe-promoter matched biological meanings.

## DISCUSSION AND CONCLUSION

We developed a computational tool named scHiMe to predict the single-cell methylation levels of cytosines and guanines (cytosines on the negative strand) of the CpGs based on single-cell Hi-C data. Graph transformer, one of the cutting-edge deep learning algorithms, was applied. We benchmarked the predictions of scHiMe from multiple perspectives and found that the predictions were accurate. There are in total two types of information input into scHiMe, in the formats of node and edge features in graphs: the DNA sequence and single-cell Hi-C data. Using the DNA sequences as input results in overall relatively accurate predictions on all cells. However, the DNA sequences used for all the cells of different cell types are the same since we used the reference genome as the input for all cells. Therefore, if we only use DNA sequences as input, the predictions on all the cells would be the same, which means the predictions cannot result in the successful classification of cells into different types. This is because if the inputs are the same and the machine learning model is the same, then the predictions/outputs will always be the same. However, our predictions result in almost perfect cell-type classifications except for the four cell types for which their single-cell Hi-C data cannot be used to successfully classify them, which indicates that the graph transformer that we have trained indeed successfully captures the patterns in the graph-structured data and our predicted DNA methylations keep the cell-to-cell variability and cell-type-specific characteristics that also exist in the single-cell Hi-C data. Moreover, the successful performance of our tool indicates that the single-cell 3D genome structure does have an influential relationship to single-cell DNA methylation, although, from this single study, we are not certain about which influences which or how one of them impacts the other.

**Key Points**

- Graph transformer can accurately predict base-pair-specific single-cell DNA methylation levels based on single-cell Hi-C data.
- The predicted methylation levels maintain the cell-to-cell variability and cell-type-specific characteristics existing in the single-cell Hi-C data.
- The genomic regions that have single-cell Hi-C contacts or are spatially proximate in the 3D space may share similar single-cell methylation levels.

## Supplementary Material

Suppl_doc_revise_5_bbad223

## Author Biographies

**Hao Zhu** is a Ph.D. student in the Department of Computer Science at the University of Miami.

**Tong Liu** is an Assistant Scientist in the Department of Computer Science at the University of Miami.

**Zheng Wang** is an Associate Professor in the Department of Computer Science, Department of Biology and Sylvester Comprehensive Cancer Center at the University of Miami.
