# Supplementary material for: scHiMe: predicting single-cell DNA methylation levels based on single-cell Hi-C data
Source: Brief Bioinform. 2023 Jun 10;24(4):bbad223. doi: 10.1093/bib/bbad223 (PMC10359091; doi:10.1093/bib/bbad223)
Supplement: Suppl_doc_revise_5_bbad223 [file suppl_doc_revise_5_bbad223.pdf]

Supplementary document for

scHiMe: Predicting single-cell DNA methylation levels based on single-cell Hi-C data

Hao Zhu, Tong Liu, and Zheng Wang*

Department of Computer Science, University of Miami, 1364 Memorial Drive, Coral Gables, FL, 33124, USA

***To whom correspondence should be addressed.**

1. Methods

1.1 Data sets

We applied the same quality control as in research (Lee, et al., 2019), based on which we only kept the cells that had total non-clonal reads > 500,000, global mCCC < 3, total autosomal cytosines covered < 100M, and total long-range (>10,000 bp) cis contacts > 5000. Additionally, the total long-range cis contacts of each chromosome need to be larger than x, where x is the length of a chromosome at Mb resolution.

1.2 Building meta-cells for single-cell Hi-C data

For each chromosome of every cell, we built a promoter-promoter contact matrix based on the single-cell Hi-C contacts of that cell (not meta-cell). Each promoter region here was defined as containing 50 kb upstream and 50 kb downstream of the TSS plus the TSS of a gene. Notice that a promoter was defined as a 1 kb region upstream of a TSS for methylation meta-cell and prediction (details will be discussed in later sections), and here we made each promoter region larger. This is because in this way more single-cell Hi-C contacts can fall in the promoter regions. In other words, having the regions too small will result in few Hi-C contacts between the regions, which will not adequately define the graph topology of the promoter-promoter interaction networks. A contact was considered to exist between two promoters if one end of a Hi-C read-pair was located inside a

promoter region and the other end was located inside another promoter region.

We performed the incremental principal component analysis function (IPCA) using the sklearn package (Pedregosa, et al., 2011) on the flattened promoter-promoter contact matrix of each chromosome and then obtained the top 30 principal components. We tested different numbers of principle components and found 30 resulted in the minimal explained variance, see supplementary Fig. S6. After concatenating the 30 principal components of 23 chromosomes (Y-chromosome not included as it contains only 63 promoters and most of the promoters do not have single-cell methylation data available), we obtained 690 principal components for each cell. We repeated the IPCA on the 690 principal components and further obtained another top 30 principal components. After that, we used these 30 components to compute the Euclidean distance between each pair of cells. Finally, we picked up the 20 nearest neighboring cells and then combined them with the central cell to generate the meta-cell for the target cell. The Hi-C contacts in the meta-cell were used to define edges in the promoter-promoter spatial interaction network.

For the cells used for blind test or benchmarking, we considered the type of each cell unknown information but the number of total cell types in the testing data set known information because most of the biological labs would have known how many and what cell types they have used in their experiments. Because of these, we clustered all the cells in the test dataset and then generated the meta-cell for each cell only using the other cells in the same cluster. We executed scHiCluster (Zhou, et al., 2019) on the intrachromosomal Hi-C contact pairs of all the cells at 1 Mb resolution. The results of scHiCluster are $n \times (n-1)$ components, where n is the number of cells and $n-1$ is the number of components for each cell. We used the spectral clustering function from the sklearn package (Pedregosa, et al., 2011) to cluster all the cells by labeling the n cells based on the output components of scHiCluster with the arguments of affinity = nearest_neighbors and cluster = 4. We used all of the components generated from scHiCluster to compute the Euclidean distance between all cell pairs. After that, we selected the 20 nearest neighboring cells in the same cluster of the target cell and then combined the Hi-C contacts of these 20 neighboring cells with Hi-C contacts of the target cell to generate the aggregated Hi-C contacts for the target cell.

1.3 Building meta-cells for single-cell DNA methylation data

The single-cell methylation data that we have used provide the number of methylated reads and the total number of reads for individual CpG. We calculated an overall methylated ratio for each promoter of a cell by dividing the sum of the methylated reads (only on CpGs) available for the promoter by the total number of reads (only on CpGs) available for the promoter. Some of the promoters in some of the cells have no methylation data at all, that is, none of the CpGs in the promoters have any reads available or the promoters do not contain any CpG. For those promoters, we computed the mean overall methylated ratios based on all of the other promoters from the same cell and used that mean value as the overall methylated ratio for each of those promoters that did not have methylation data available.

We calculated a variance value for each promoter based on the overall methylated ratios of the promoter in all cells. After that, we selected 5000 promoters with the highest variance values and performed IPCA on their overall methylated ratios. For each promoter, we obtained the top 30 components, which were used to calculate the Euclidean distance between every cell pair. Based on the Euclidean distances, we chose the 20 nearest neighboring cells and then combined them with the target cell to generate the meta-cell.

The aggregated methylation levels of the meta-cells were used as the true or target methylation levels of the target cell. For example, for base pair 1 in the promoter p_1, five of the 21 cells in the meta-cell have methylation data available, that is, each of the five cells provides both the number of methylated reads on that base pair and the total number of reads on that base pair. The true methylation level or the target value for base pair 1, which was a value in the range of [0, 1], was calculated as the total number of methylated reads from the five cells divided by the total number of reads from the same five cells. If none of the 21 cells in the meta-cell had methylation levels available on a base pair, then we labeled -1 as the target value for the base pair. If a nucleotide base in a promoter was not the cytosine (C) of a CpG, we used -1 as the target value for the base pair, unless the nucleotide base was guanine (G) and its complementary base was the cytosine (C) of a CpG. In that case, we used the methylation level of the complementary base as the target value of the base pair.

In summary, each promoter has 1000 target values, one for each base pair in the promoter region. Notice that the final predictions from scHiMe also contain 1000 values for each promoter (one for each base pair), and the predictions are provided based on the reference genome, which is the DNA sequence of the positive strand.

Therefore, the predicted methylation level on a G of a CpG dinucleotide can be considered as the prediction made for its complementary C in the negative strand.

1.4 Node and edge features

The first edge feature contains 21 integers, which are the numbers of Hi-C contacts, between the two promoters that the edge connects, in the target cell and 20 neighboring cells in the meta-cell. The second edge feature is the average number of occurrences of nonzero Hi-C contacts in the meta cell. For example, if there are only two nonzero Hi-C contact values among 21 Hi-C contact values in the meta-cell, which are 3 and 1 as shown in the example in Fig. 1, then the second edge feature is 0.095, which is calculated as 2 / 21. The third feature is calculated as $\frac{1}{5}$ of the base-10 logarithm of the genomic distance between the two promoters that are connected by the edge. The genomic distance here is at a resolution of 1 bp. In total, the edge feature for each edge consists of 23 values.

1.5 Training, validation, and blind test

We used the Adam algorithm (Paszke, et al., 2019) as our optimizer to train the graph transformer. We used the function ReduceLROnPlateau in the torch.optim package (Paszke, et al., 2019) with the parameters mode=min, patience = 5, and factor = 0.1" to automatically reduce learning rates. The learning rate was set to 0.001, and the batch size was set to 1. The training loss was calculated as the average absolute differences between the predicted methylation levels and the target or ground-truth methylation levels. The training loss was calculated on the base pairs that did not have -1 as the target values.

We used the Vip and Sst cell types from the first data set to generate the validation data. There are 2254 graphs for validating the graph-transformer model. We terminated the training process when the validation loss stopped decreasing for five consecutive epochs or the number of epochs reaches 100. The model with the smallest validation loss was selected as the final model and benchmarked on the blind test data sets.

We used Endo, L23, MG, and OPC cell types from the first and second data sets, which corresponded to 7544 and 24219 graphs, respectively, to generate the blind test data. From the third data set, we also chose the

top 100 cells having the highest number of Hi-C contacts from each of the K562, HAP1, and HeLa cell types to create the extra blind test data.

1.6 scHiMe-exon1

We analyzed the lengths of the first exons of all genes in the GENCODE human annotation version 19 dataset and found that 97% of the first exons had lengths within 2000 base pairs to the transcription start site (TSS). Since the first intron usually exists between the TSS and the first exon, we believe the 2000 bp range should cover almost all of the first introns. Based on this observation, we defined a node as a DNA sequence consisting of 1999 base pairs upstream and 500 base pairs downstream of the transcription start site (TSS), plus the TSS itself. We then used a 6-mer DNABERT to extract node features for each node. For edge features, we used scHiMe-promoter's edge features for scHiMe-exon1. We trained the model of scHiMe-exon1 using the same training parameters as scHiMe-promoter and generated training, validation, and blind test data using the same cell type as scHiMe-promoter.

1.7 scHiMe-whole-genome

We first partitioned the entire genome into non-overlapping segments of 1000 base pairs and then for each segment calculated the ratio of base pairs that have true methylation data available. We plotted a histogram, see supplementary Fig. S7, showing the distribution of the numbers of segments in each cell that have this ratio > 0.1. We picked up a representative cell that existed in the middle range of the histogram and then used all of its segments that have > 0.1 ratios to be the template segments, which means for all of the other cells the same segments will be used for generating training, validation, and blind-test datasets, even though their numbers of segments with > 0.1 ratios may be smaller than the representative cell, or the same segments in some other cells may not have the ratio > 0.1. In this way, our data set consists of various types of segments or cells, regardless of their true methylation ratios. We generated node features using a 6-mer DNABERT model and built edge and edge features using the same methods as for scHiMe-promoter. We created training, validation, and blind test datasets using the same cell type as scHiMe-promoter. We trained the scHiMe-whole-genome model using the

same training parameters as scHiMe-promoter, except for the learning rate which was set to 0.0001.

1.8 Evaluation

1.8.1 Naive predictors

Since there is no other published tool to compare performances, we built two naive predictors. The predictions for a promoter from naive-predictor-1 are the average methylation levels gathered from the radius-one neighboring promoters in the promoter-promoter spatial interaction network. For naive-predictor-2, we defined a target region for each of the base pairs in the target promoter, which contains the target base pair, 50 bp upstream of the target base pair, and 50 bp downstream of the target base pair. All of the single-cell Hi-C contacts with one end falling into the target region were collected. For each of the other ends in these Hi-C contacts, we defined an in-contact region, which contains the in-contact base pair that formed the Hi-C contact with the target base pair, 50 bp upstream of the in-contact base pair, and 50 bp downstream of the in-contact base pair. An average methylation value was calculated based on all of the available ground-truth per-base-pair methylation levels within all of the in-contact regions, which was considered the final prediction from naive-predictor-2 for the target base pair.

1.8.2 Cell classification and its evaluation

Besides clustering cells using all promoters, we also benchmarked the performances of clustering cells using individual promoters or individual CpGs because we believed this provided biologists with more information about which promoters or CpGs and their methylation levels were important in differentiating cells to different cell types. In these cases, the same methodologies for clustering, calculating ARI scores, and visualization were applied with the only difference being that the methodologies were applied on the methylation levels of one or selected promoter(s), cytosine(s), or guanine(s).

1.8.3 Importance of individual CpGs in promoters for clustering cells

We used the predicted methylation levels on all of the CpGs, including both cytosines and guanines, from all of the promoters genome-wide as the features. In other words, each training example is for one cell with the target value as the true cell type for that cell, followed by 1443574 predicted methylation levels. We used all of the

1053 cells in the four cell types in data set 2 to generate the training and validation data set for the random forest: 80% of the cells were used for training, and 20% for validation. We tested different values for the number of trees including 100 and 1000, which resulted in classification accuracies of 0.857 and 0.995, respectively. Based on these, the feature importance was generated based on the random forest model that consisted of 1000 trees. Each of the cytosines and guanines in all of the CpGs had a feature importance value output from the random forest algorithm, which indicated the importance of each cytosine or guanine when used to classify cells into the four cell types.

1.8.4 Collective Influence algorithm

We applied the collective influence (CI) algorithm (Morone and Makse, 2015) on the promoter-promoter spatial interaction networks built on data set 2. A promoter-promoter spatial interaction network was built for each chromosome of a cell, and all of the promoter-promoter interaction networks of a cell were saved in one file and input into the CI algorithm for finding network influencers. The nodes that had no edge connecting to any other nodes were removed. A list of influencers ranked from the most significant to the least significant influencers was outputted from the CI algorithm for each cell.

To find the relationship between the ranking position of each promoter in the influencer list and the ARI score of using the promoter to classify cells, we defined two ways of calculating the average ranking position in the influencer list for each promoter, which are named avg_inf_rank1 and avg_inf_rank2. For each promoter in avg_inf_rank1, if its ranking position for a cell was smaller than t, then this original ranking position was used. If its ranking position for a cell was larger than t, or if the promoter was not even included in the ranking list for a cell, then $t + 1$ was used as its ranking position for that cell. Two values for threshold t were tested, which were 10 and 17822. When using 10, we focused on the cases where a promoter was among the top 10 most significant influencers. When using 17822, we included all possible ranking positions since the longest ranking list contained 17822 influencers. When using the threshold 17822, we only used the cells that had at least 16000 influencers output from the CI algorithm.

For each promoter in avg_inf_rank2, if its ranking position for a cell is smaller than t, then this ranking position was kept. We gathered all of the ranking positions for the promoter that meet this criterion and then

calculated the average ranking position for that promoter. This process was repeated for all of the promoters. The thresholds t that we tested were 10 and 17822. When using 17822 as the threshold, we only used the cells that had at least 16000 influencers output from the CI algorithm.

2. Results

2.1 Cell classification and its evaluation

Supplementary Fig. S2A shows the ARI-all-data scores of clustering cells based on the predicted methylation levels of all cytosines and guanines of the individual or selected promoters on data set 2. We used the scHiMe-predicted methylation levels for each promoter to cluster cells and then ranked the promoters based on the ARI-all-data scores. It can be found that using only the first-ranked promoter resulted in an ARI score of 0.664 and using the top 32 promoters brought the ARI score to 0.898. However, using more than the top 50 promoters did not further improve the ARI scores. The ARI scores and the visualizations of clustering results on the top two components for the No. 1-200 and top 1-200 promoters can be found in supplementary data.

We further tested the performance of clustering cells using the scHiMe-predicted methylation level of each cytosine or guanine of a CpG on data set 2. Supplementary Fig. S8 shows the distribution of the number of cytosines and guanines having various values of feature importance. We ranked all of the cytosines and guanines based on their feature importance and clustered cells using the top n cytosines or guanines. The ARI-all-data scores for the top 1-1000 were plotted in supplementary Fig. S2B. It can be found that when the top 28 cytosines or guanines were used, the ARI score reached 0.572 and remained there even when the number of cytosines or guanines went up to 1000. The ARI-all-data scores and the visualizations of the top two t-SNE components of the top 1-1000 cytosines or guanines can be found in the supplementary data.

2.2 Performance from the perspective of network influencer

We also benchmarked the performance of scHiMe from the perspective of network influencers. Supplementary Fig. S9 shows the distribution of the number of influencers found for all the cells in data set 2. It can be found

that 85% of the cells have from 14007 to 17822 influencers detected from their promoter-promoter spatial interaction networks. We studied the degrees of the top 10 influencers for all cells in data set 2, see the distribution in supplementary Fig. S5 A. We did find higher mean degree values for the first-ranked and second-ranked influencers, which are 7.374 and 6.964, respectively. For the influencers ranked from the third to the tenth, we find that they have similar mean degree values.

Supplementary Fig. S5 B shows the evaluation metrics for the influencers ranked from the first to the fourteenth on data set 2. We did not find a trend of increasing or decreasing for all of the evaluation metrics with the increase of the influencer ranking positions.

We further investigated the relationship between influencer significance and the feature importance of the cytosines and guanines in CpGs on data set 2, see supplementary Fig. S10 and S11. Similar to the five evaluation metrics, we did not find an obvious relationship between influencers and feature importance.

We studied whether the most significant influencers resulted in higher ARI-all-data scores when the predicted methylation levels on the influencers were used to cluster cells into different cell types. Supplementary Fig. S5 C shows our benchmark results on all of the cells but only considers the top 10 most significant influencers. Supplementary Fig. S5 D illustrates the results on the cells that have at least 16000 influencers detected, which has a Pearson correlation of 0.098 between influencer ranking positions and ARI-all-data scores. In summary, the results from supplementary Fig. S5 C and D do not show a strong correlation or relationship between the influencer ranking positions and ARI scores. The average ranking positions in supplementary Fig. S5 C and D were calculated using avg_inf_rank1. Supplementary Fig. S12 shows the distributions of the number of influencers with various avg_inf_rank1 values associated with supplementary Fig. S5 C and D, respectively. Supplementary Fig. S13 shows the results using avg_inf_rank2 on data set 2, which indicates the same conclusions.

Last but not least, we picked up the influencers that were first-ranked in the influencer lists of all cells and counted the number of occurrences of each promoter as the first-ranked influencer among all cells. The promoters that have the same number of occurrences were used to cluster cells and the ARI-all-data scores were calculated. Besides the first-ranked influencers, we also conducted the same experiments for the second-ranked,

third-ranked, fourth-ranked, and fifth-ranked influencers, see Supplementary Fig. S14 A-E. No significant Pearson correlation or relationship was found between the ARI-all-data scores and the number of occurrences. We also picked up the top five most occurred influencers for influencer ranking positions 1 to 93 (the shortest influencer list contained 93 influencers) and used the top five influencers to clustering cells, see Supplementary Fig. S14 F, from which the same conclusion was drawn.

3. Figures

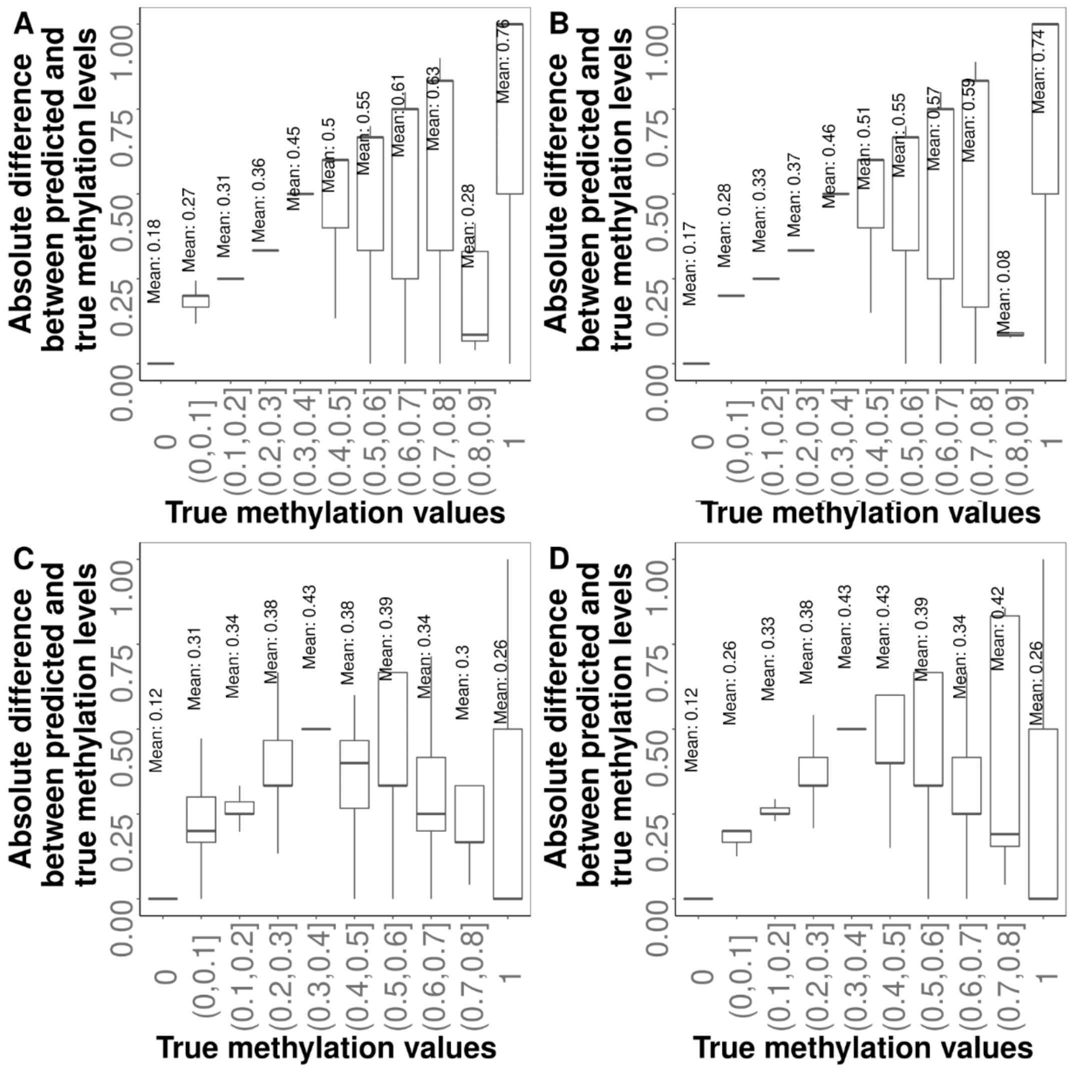

Figure S1. A-B. The absolute differences between the naive predictor 1-predicted and true methylation levels on the blind-test cells in data sets 1 and 2. **C-D.** Similar to **A-B**, but on naive predictor 2-predicted methylation levels.

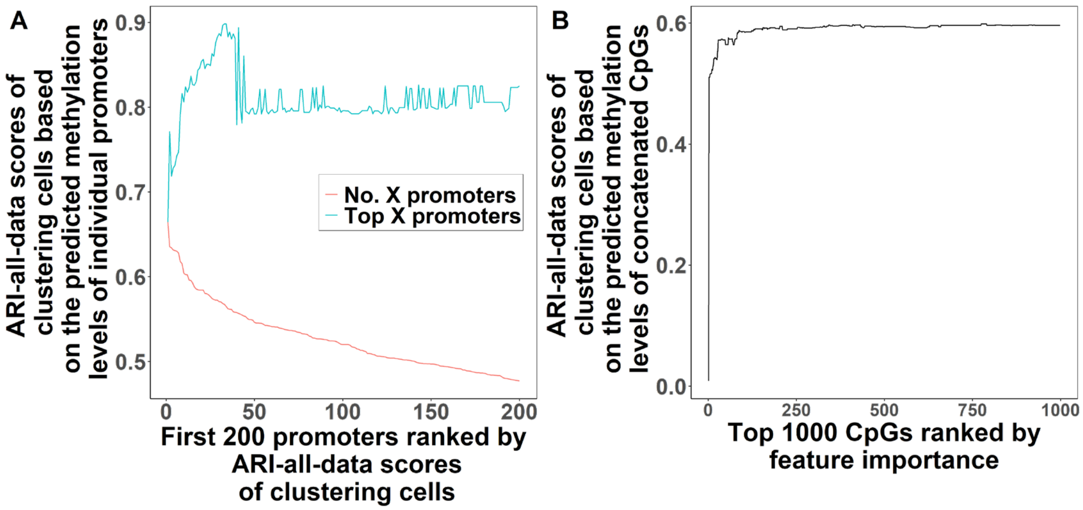

Figure S2. A. The ARI-all-data scores of clustering on the first 200 promoters ranked by ARI scores. The first-ranked promoter was labeled as No. 1, the second-ranked No. 2, and so on. The red line indicated the ARI-all-data scores when only No. n $(1 \leq n \leq 200)$ promoter was used, and the values of n were shown as the X-axis. The blue line indicated the performance when top n promoters were used, which meant when all of the first-ranked, second-ranked, …, and the n-th-ranked promoters were used. **B.** The ARI-all-data scores of the top 1000 CpGs that were ranked by feature importance.

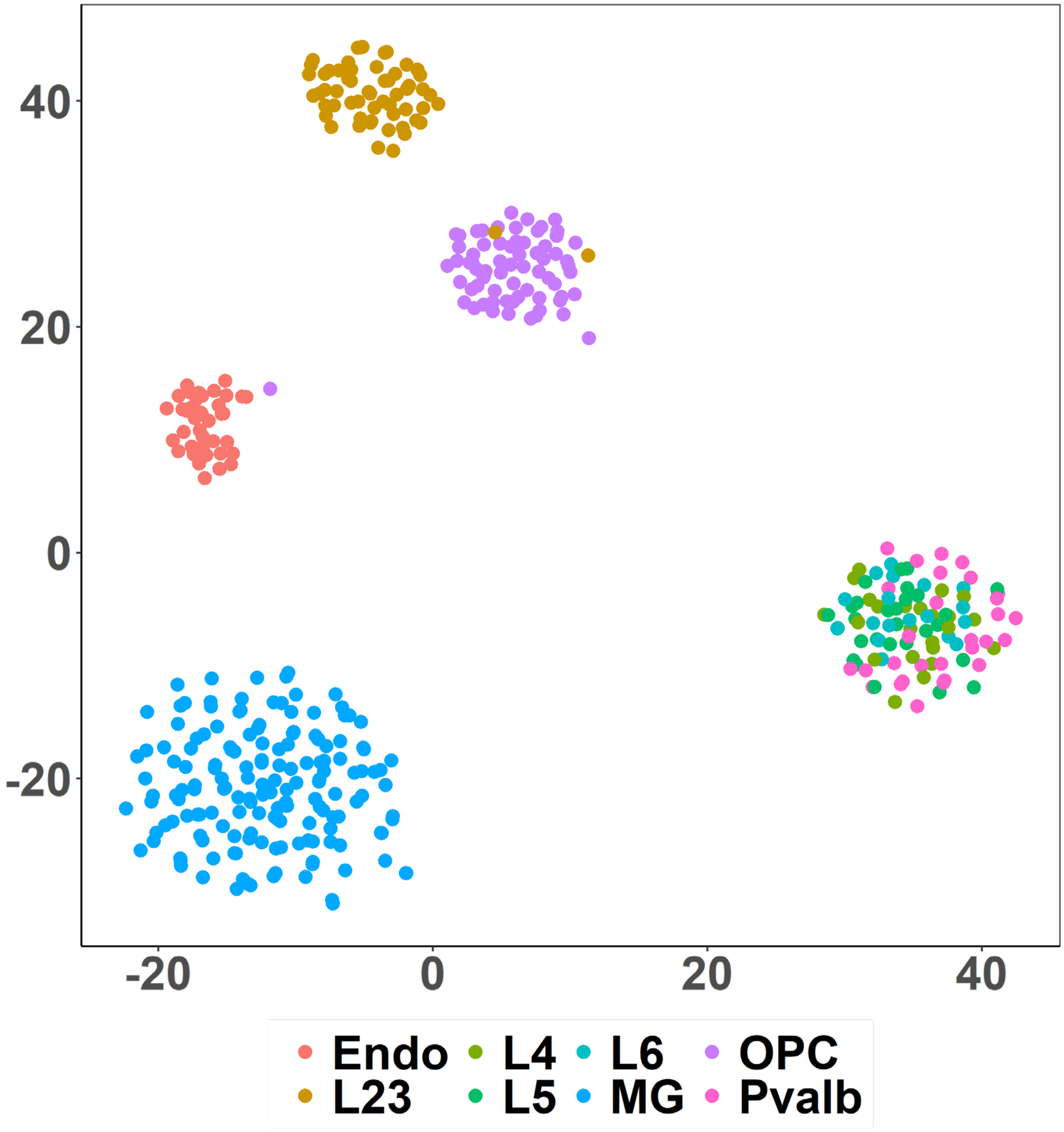

Figure S3. The Clustering results based on the scHiMe-promoter predictions on eight cell types in data set 1.

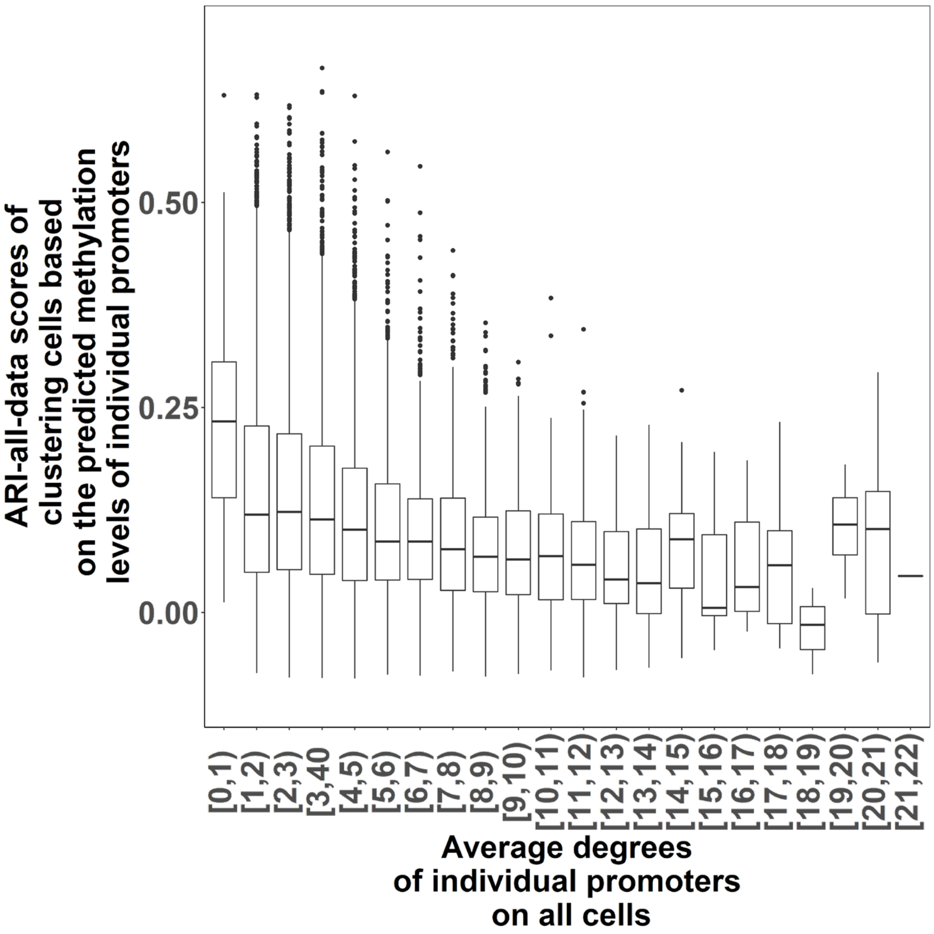

Figure S4. The ARI-all-data scores of clustering cells based on the scHiMe-promoter-predicted methylation levels of the individual promoters of different average degrees.

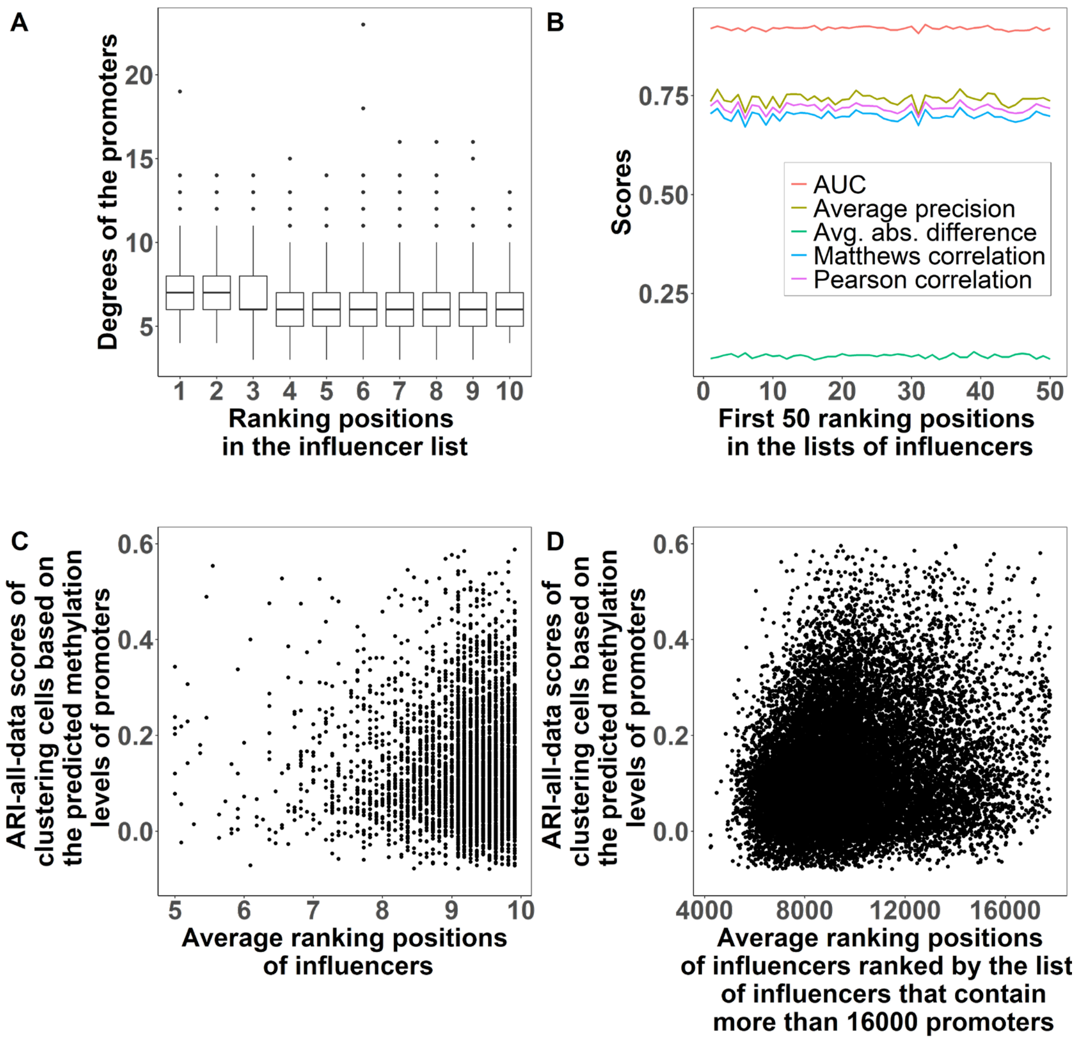

Figure S5. A. The distribution of the degrees of the top 10 influencers for all cells in data set 2. **B.** Evaluation matrices for top 50 influencers on data set 2. **C.** ARI-all-data scores of clustering cells based on the predicted methylation levels on all cells based on the top 10 most significant influencers (promoters). **D.** Similar to **C**, but on the cells that have at least 16000 influencers (promoters) in the list.

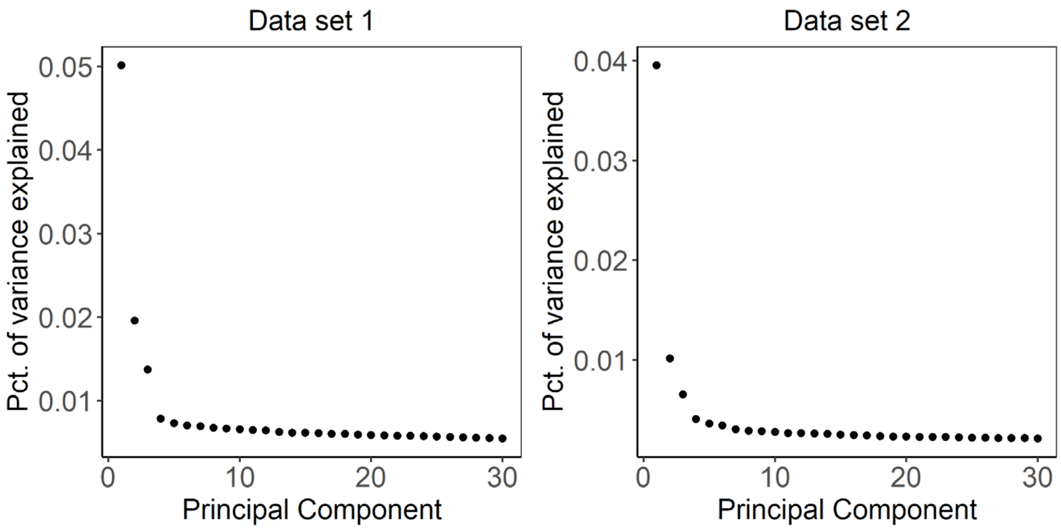

Figure S6. The percentage of variance in the data explained by each principal component in the principal component analysis, sorted from highest to lowest.

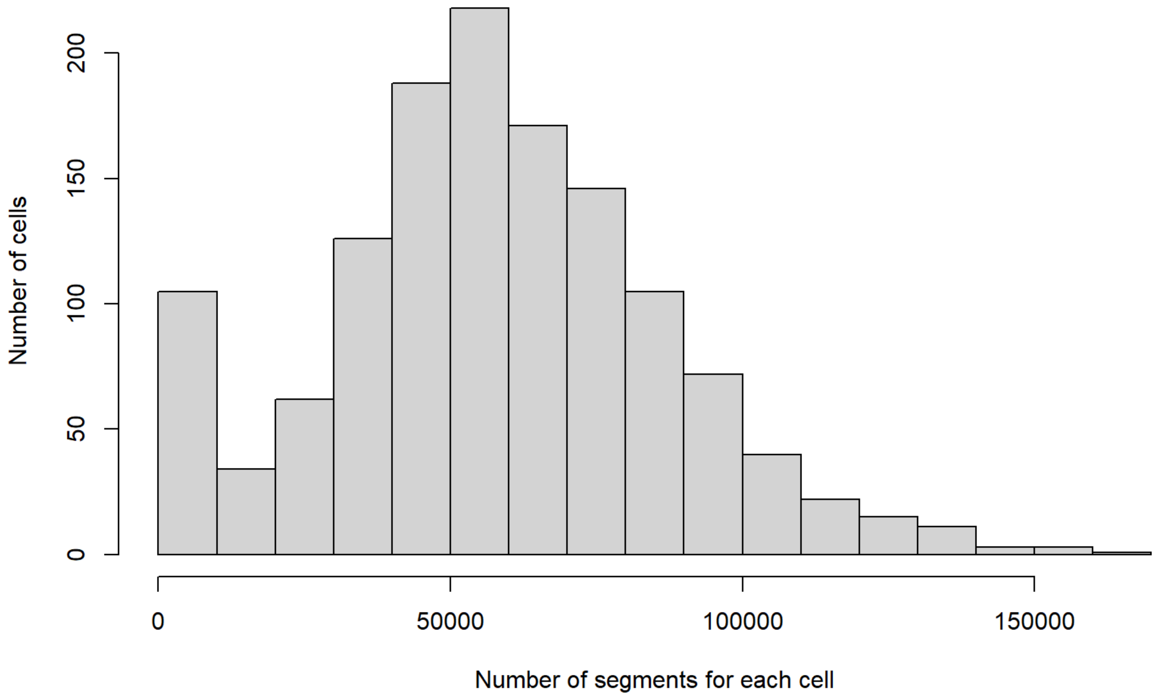

Figure S7. The distribution of the numbers of segments in each cell that have true methylation ratio > 0.1

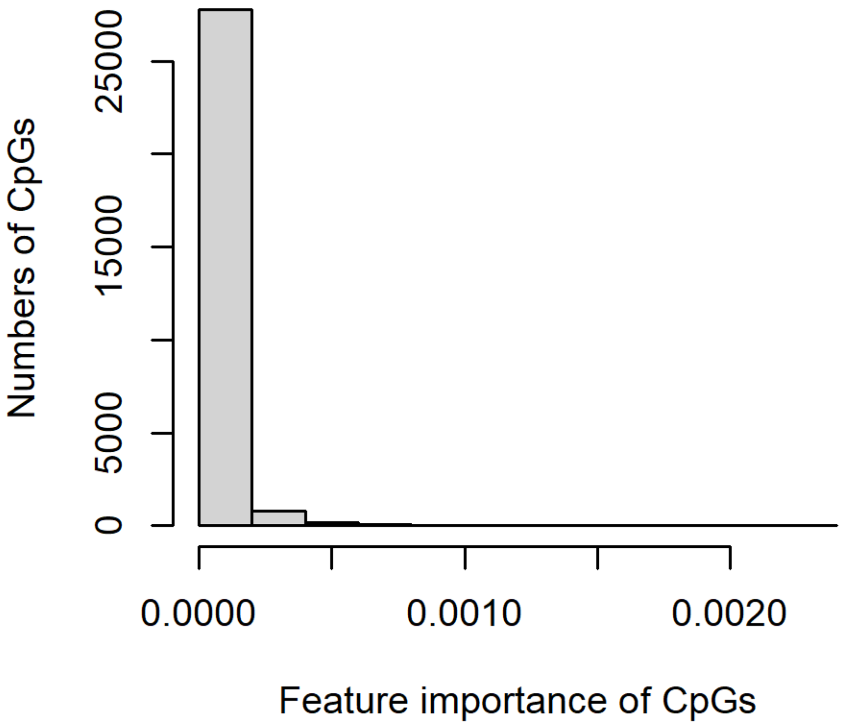

Figure S8. The distribution of the numbers of cytosines and guanines having various values of feature importance.

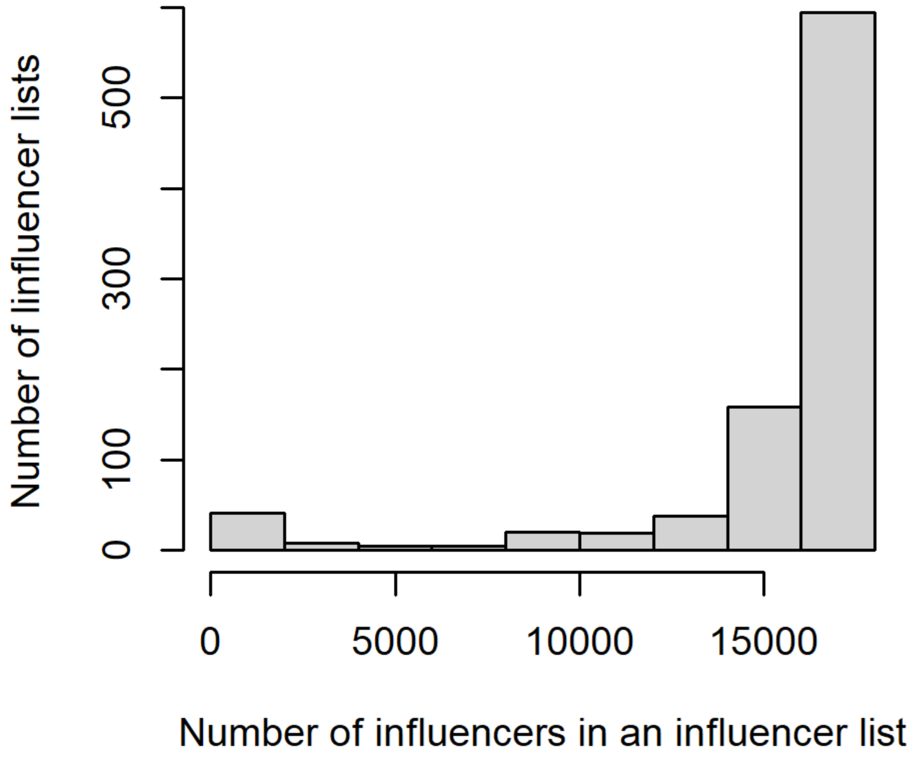

Figure S9. The distribution of the numbers of influencers found for all the cells in data set 2.

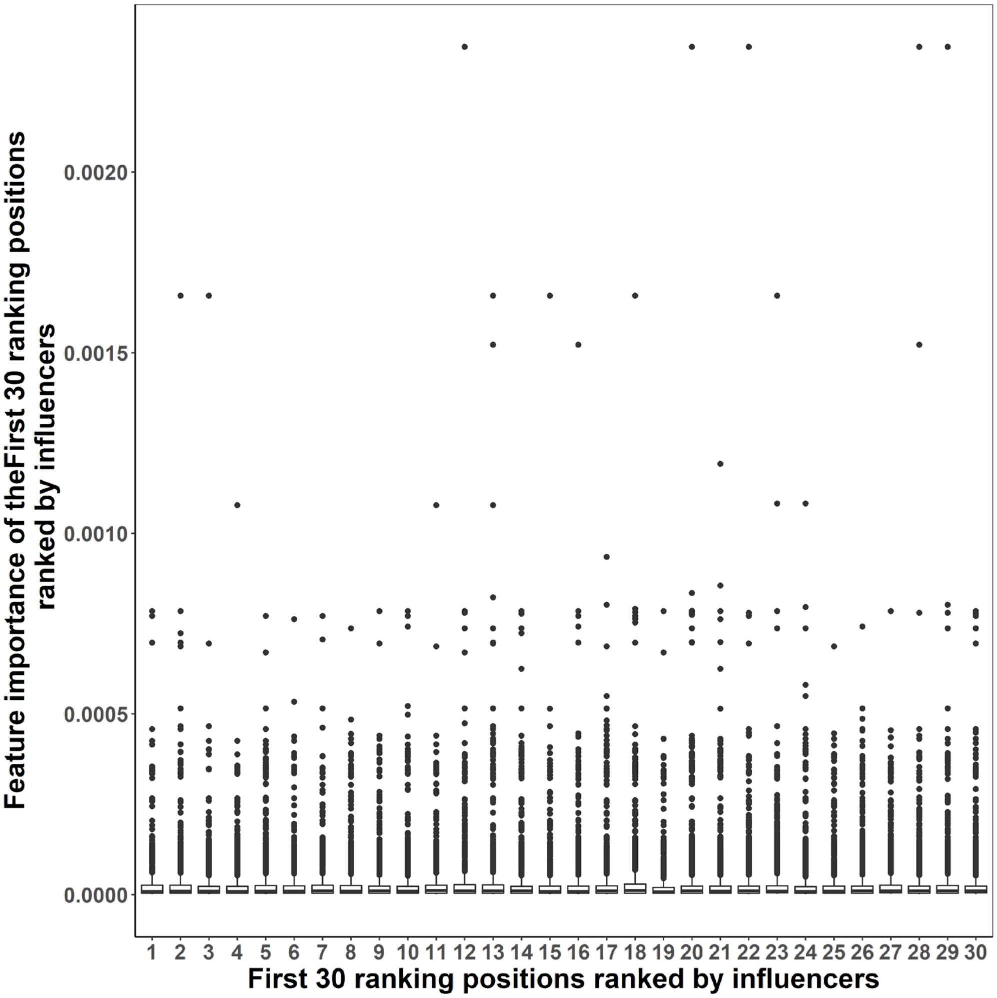

Figure S10. The feature importance of the Cs and Gs of CpGs that exist in the promoters at the same ranking positions. The ranking positions are from all the lists of influencers.

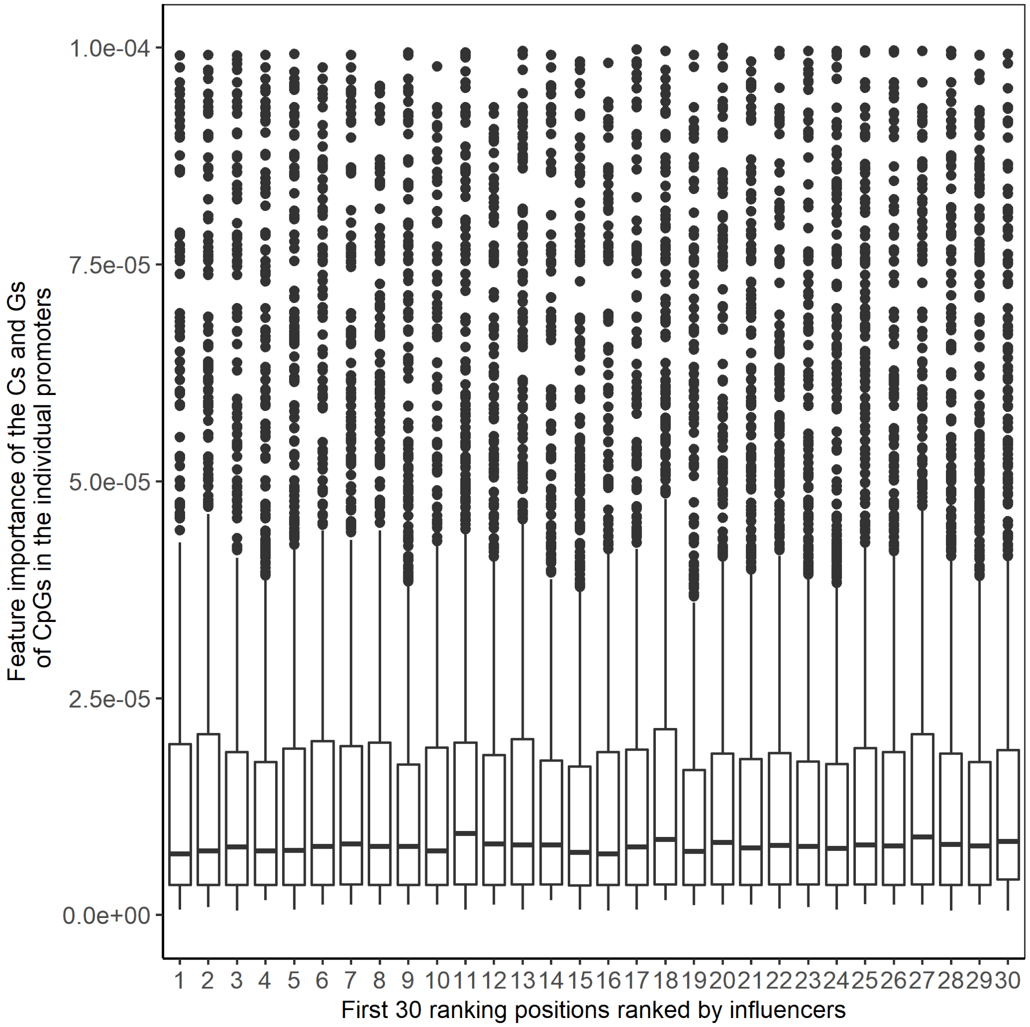

Figure S11. The feature importance of the CpGs that exist in the promoters at the same ranking positions from all the lists of influencers. This figure only shows a part of the feature importance from 0 to 0.0001

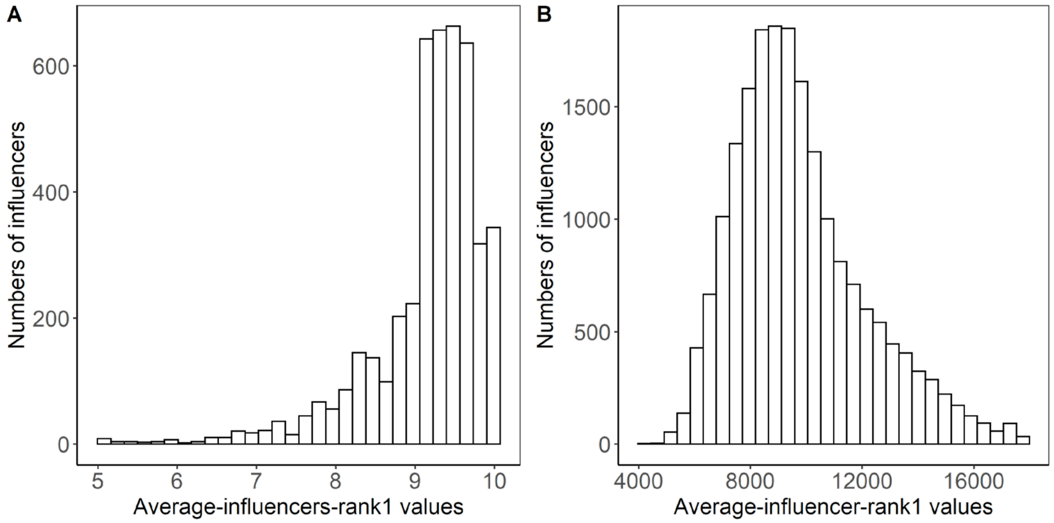

Figure S12. A-B. The distribution of the number of influencers with various average-influencer-rank1. **A**. The influencers are top 10 most significant influencers from all cells. **B.** The influencers are from the cells that have at least 16000 influencers detected.

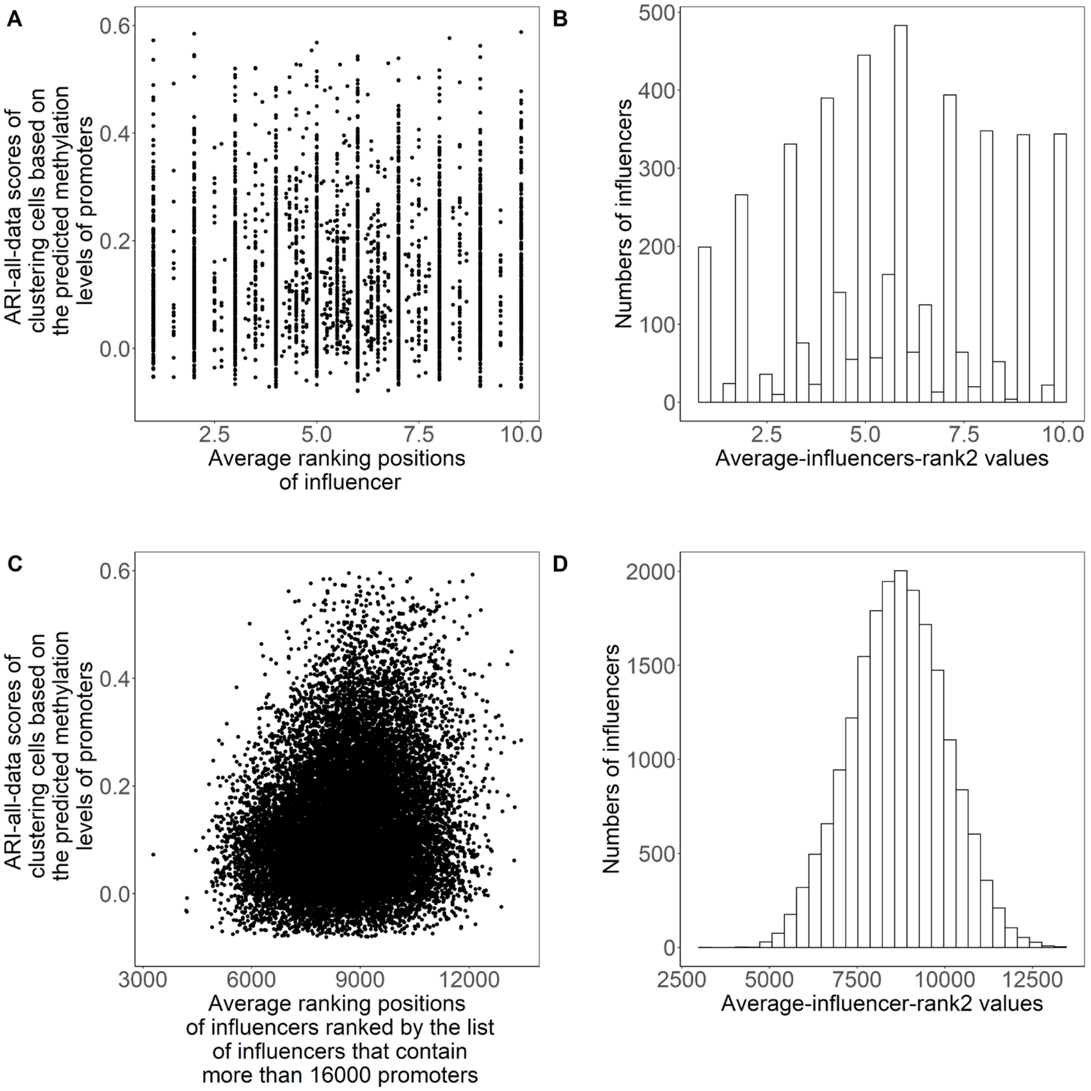

Figure S13. A. The ARI-all-data scores of clustering cells based on the predicted methylation levels on all cells based on the top 10 most significant influencers (promoters). **B.** The distribution of the number of influencers with average-influencer-rank2. The influencers are top 10 most significant influencers form all cells. **C.** Similar to **A**, the influencers are from the cells that have at least 16000 influencers (promoters) in the list. **D.** The distribution of the number of influencers with average-influencer-rank2. The influencers are top 10 most significant influencers form all cells. The influencers are from the cells that have at least 16000 influencers detected.

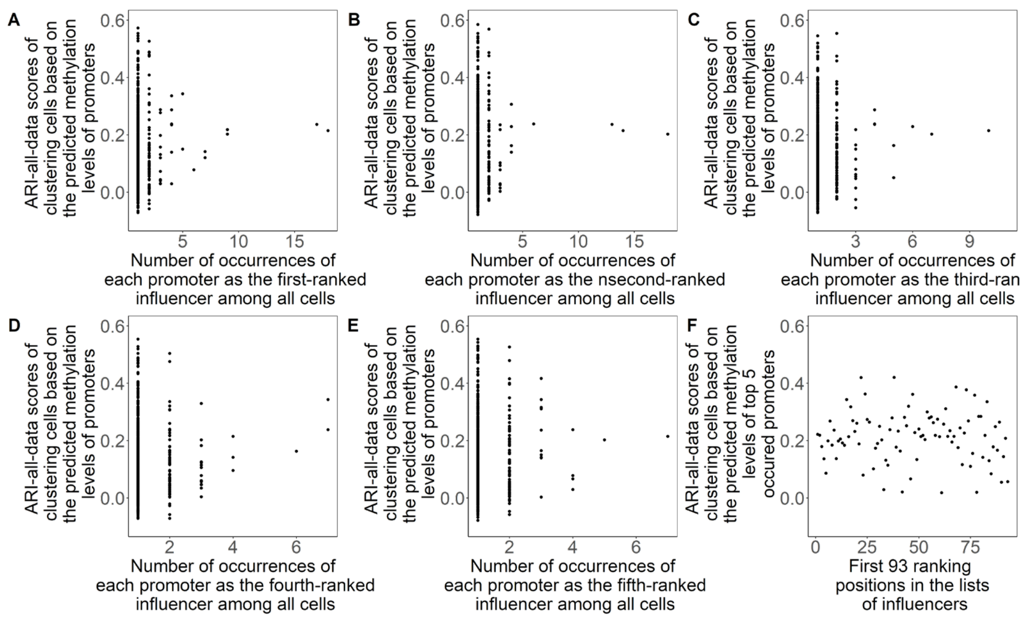

Figure S14. A-E. The ARI-all-data scores of clustering cells based on the predicted methylation levels of influencers (promoters) from No. 1 to No. 5 positions in the lists of influencers. **F.** The ARI-all-data scores of clustering cells based on predicted methylation levels of top 5 occurred promoters.

4. Tables

Table S1. The number of training, validation, and blind testing cells used in datasets 1 and 2.

Prediction method	Number of cells used	Data set 1	Data set 2
scHiMe-promoter	Training	946	Did not use
scHiMe-promoter	Validation	96	Did not use
scHiMe-promoter	Blind-test	328	1053
scHiMe-promoter-mixed	Training	473	473
scHiMe-promoter-mixed	Validation	48	48
scHiMe-promoter-mixed	Blind-test	164	164
scHiMe-exon1	Training	226	Did not use
scHiMe-exon1	Validation	96	Did not use
scHiMe-exon1	Blind-test	328	328
scHiMe-whole-genome	Training	376	Did not use
scHiMe-whole-genome	Validation	50	Did not use
scHiMe-whole-genome	Blind-test	328	328

Table S2. The evaluation results for generating the node features by using 4-mer and 5-mer DNABERT models for scHiMe-promoter.

mers	Data set	PCC	Average precision	MCC	AUC	Mean abs. error
4-mer	Data set 1	0.718	0.731	0.695	0.908	0.105
4-mer	Data set 2	0.689	0.717	0.669	0.895	0.110
5-mer	Data set 1	0.742	0.790	0.708	0.941	0.102
5-mer	Data set 2	0.736	0.789	0.702	0.935	0.103

Table S3. The number of examples used for training, validation, and blind-test for three scHiMe-predictors

	Number of scHiMe-promoter examples	Number of scHiMe-exon1 examples	Number of scHiMe-whole-genome examples
Training	21758	5198	8648
Validation	2208	2208	1150
Blind-test data set 1	7544	7544	7544
Blind-test data set 2	24219	7544	7544

Table S4. The evaluation results of scHiMe-promoter for each cell type in data set 1.

Cell types	Data set	PCC	Average precision	MCC	AUC	Mean abs. error
Endo	Data set 1	0.721	0.765	0.699	0.934	0.097
L23	Data set 1	0.728	0.773	0.706	0.936	0.097
MG	Data set 1	0.723	0.768	0.701	0.934	0.095
OPC	Data set 1	0.718	0.763	0.697	0.933	0.099

Table S5. The number of nodes in chromosome 1 used in three scHiMe-predictors

Number of promoters in Chr1 used in scHiMe-promoter	Number of nodes in Chr1 used in scHiMe-exon1	Number of nodes in Chr1 used in scHiMe-whole-genome
2021	2021	4840

Table S6. The evaluation results of scHiMe-promoter on blind test data with eight cell types in data set 1.

Prediction method	Data set	PCC	Average precision	MCC	AUC	Mean abs. error
scHiMe-promoter	Data sets 1	0.734	0.774	0.712	0.937	0.095

Table S7. The top 20 promoters' gene names with the highest ARI-all-data scores based on scHiMe-promoter predicted methylation levels in the data sets 1 and 2.

ARI-all-data rank	Data set 1	Data set 2
1	CD1B	CALML4
2	OR10T2	CNOT11
3	KCNIP3	MGMT
4	FAM47E	TAF9B
5	DDX60	CT47A7
6	HIST1H1A	CYB561
7	SMOC2	CT47B1
8	ATP6V1H	SERAC1
9	EIF3H	ARMCX5
10	RP11-451M19.3	RP11-383H13.1
11	MGMT	TPRXL
12	PKP2	PTPN14
13	DPY19L2	ALG13
14	ZFC3H1	C15orf61
15	THAP2	ANKRD20A3
16	PRKCH	FBXO21
17	ZNF98	LRIG3
18	MYH9	CERS6
19	ZNF280C	CHRDL2
20	MAGEA3	CARHSP1

Reference

Lee, D.S., *et al.* Simultaneous profiling of 3D genome structure and DNA methylation in single human cells. *Nat Methods* 2019;16(10):999-1006.

Morone, F. and Makse, H.A. Influence maximization in complex networks through optimal percolation. *Nature* 2015;524(7563):65-68.

Paszke, A., *et al.* Pytorch: An imperative style, high-performance deep learning library. 2019;32:8026-8037.

Pedregosa, F., *et al.* Scikit-learn: Machine learning in Python. 2011;12:2825-2830.

Zhou, J., *et al.* Robust single-cell Hi-C clustering by convolution-and random-walk–based imputation. 2019;116(28):14011-14018.
